# Sphenoid sinus neuroendocrine carcinoma

**DOI:** 10.1259/bjrcr.20150334

**Published:** 2016-06-08

**Authors:** Eduardo Matarolo Jayme, Tauy Pereira Morimoto, Thales Masirevic Lozano, Zélia Maria de Sousa Campos, Cláudio Campi de Castro

**Affiliations:** Department of Radiology, Faculdade de Medicina do ABC, Santo André, Brazil

## Abstract

Neuroendocrine tumours are epithelial neoplasms with predominant neuroendocrine differentiation. The nasal cavity and paranasal sinuses are rare locations for neuroendocrine carcinomas, and only a few related papers have been published in the literature to date. Here we present the case of a 64-year-old male with neuroendocrine carcinoma of the sphenoid sinus, along with the main MRI findings.

## Clinical presentation

A 64-year-old male presented to the otorhinolaryngologist with a 2-year history of a continuous, daily, frontal headache associated with left earache. The patient showed no otorrhoea, hearing loss or bleeding from the ear.

## Imaging findings

**Figure 1.**
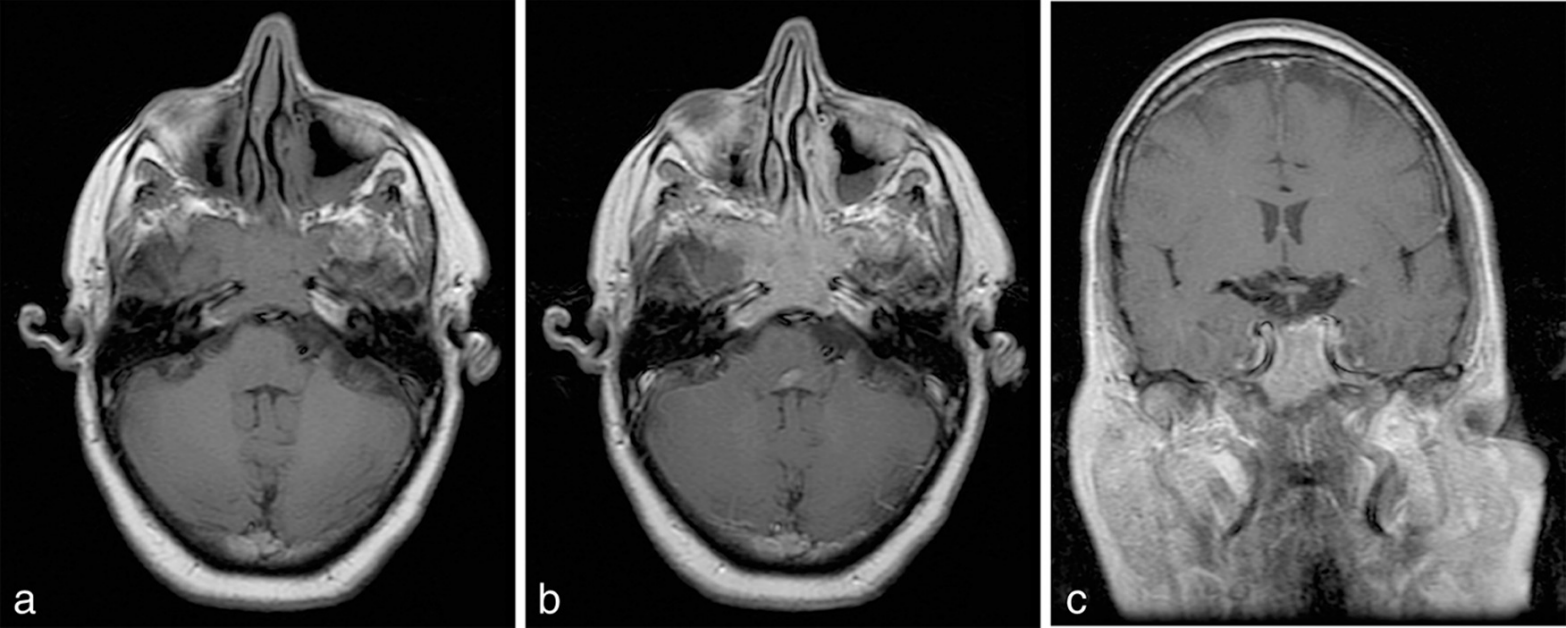
MRI scan demonstrating a mass in bilateral sphenoid sinus. (a) Axial unenhanced *T*_1_, (b) axial post-contrast *T*_1_ and (c) coronal post-contrast *T*_1_ images.

**Figure 2.**
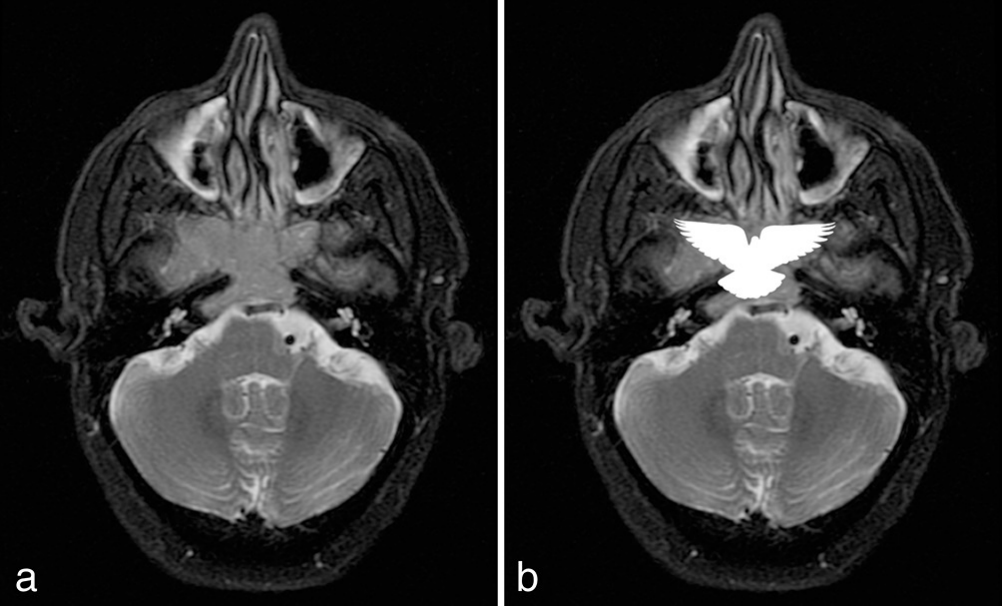
Axial fat-suppressed *T*_2_ weighted MRI sequence. (a) The lesion produces a symmetrical pattern that resembles a pigeon. (b) Schematic silhouette of a pigeon projected over the mass.

MRI revealed an isointense mass on *T*_1_ and *T*_2_ imaging, with moderate and homogeneous contrast enhancement. The lesion occupied the bilateral sphenoid sinus in a symmetrical or pigeon-like pattern, with bone destruction of the clivus and the floor of the sella, involvement of the internal carotid arteries and infiltration of the cavernous sinuses, especially on the right side (Figures 1 and 2).

## Investigation

**Figure 3.**
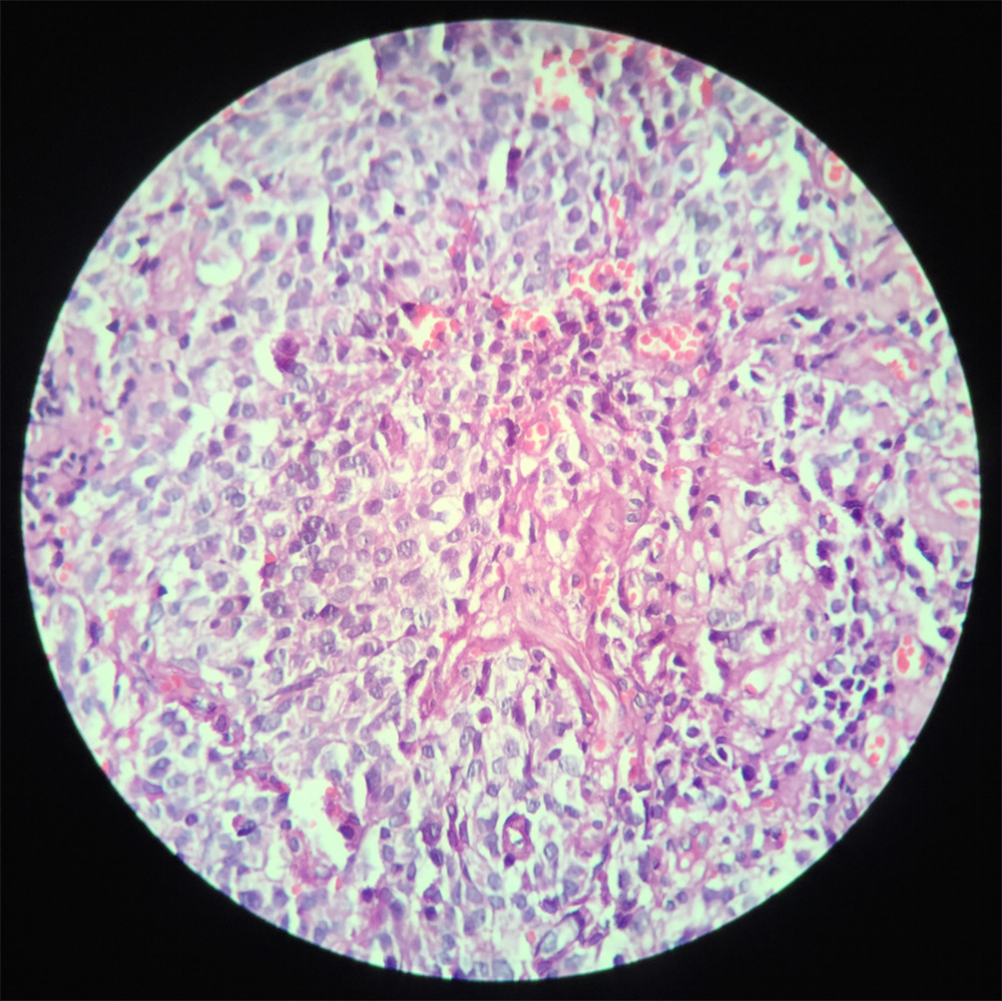
Epithelioid cells with clear cytoplasm and hyperchromatic nuclei within a vascular stroma.

Biopsy and histopathological examination revealed a poorly differentiated malignant neoplasm (Figure 3). Immunohistochemical analysis showed that the lesion was positive for cytokeratin, enolase and chromogranin, with a Ki-67 index of 10%. On the basis of these findings, neuroendocrine carcinoma (NEC) was diagnosed.

## Differential diagnosis

The differential diagnosis of sphenoid sinus NEC includes other tumours involving the nasal cavity and paranasal sinuses, such as squamous cell carcinoma, sinonasal undifferentiated carcinoma, adenoid cystic carcinoma and adenocarcinoma.^1,2^ Squamous cell carcinoma is the most frequent histopathological subtype of paranasal sinus tumour, with a prevalence of approximately 60%–75%. Other paranasal sinus neoplasms such as lymphomas, olfactory neuroblastomas and melanomas do not usually involve the sphenoid sinus.^2^

## Treatment, outcome and follow-up

The patient underwent chemotherapy with cisplatin and etoposide. Following four cycles of chemotherapy, no significant tumour regression was observed. A surgical approach was not thought to be feasible because of the extent of the lesion.

## Discussion

NECs are epithelial neoplasms with predominant neuroendocrine differentiation.^1^ Although they can be found almost anywhere in the body, the most frequent sites are the gastrointestinal tract and respiratory system.^1,3^ In the head and neck region, neuroendocrine tumours most commonly appear in the larynx.^4–6^

NECs are categorized into well differentiated (typical), moderately differentiated (atypical carcinoids) and poorly differentiated (small- and non-small-cell types). The prognosis is worse for less-differentiated neoplasms.^5^

Tumours—neuroendocrine included—of the nasal cavity and paranasal sinuses are rare, representing 0.2–0.8% of all cancers and 1–3% of head and neck carcinomas.^2,4^ Neoplasms are far less common when only the sphenoid sinus is involved, constituting approximately 2%–3% of paranasal sinus tumours.^2,7^

Sinonasal tract NEC was first categorized by Silva et al^8^ in 1982. This tumour is rare and published literature on the subject is scarce.^5,6^ It affects mainly older patients (fifth and sixth decades) and is slightly more frequent in males.^1^ The most common sites involved are the ethmoid sinuses and the nasal cavity.^6^ Distant metastases are uncommon, and mostly occur in the brain and spine.^4^ The most frequent symptoms of sinonasal neuroendocrine neoplasms include nasal obstruction, epistaxis and nasal drainage, which are non-specific, often delaying the diagnosis and appropriate treatment. The majority of patients present with advanced-stage disease.^6^

Contrast-enhanced MRI and CT scan are not usually definitive of a primary sphenoid neoplasm, although some findings may be suggestive, especially retrospectively. An MRI provides detailed information on the relationships between the tumour and the surrounding structures; therefore, this technique is superior in assessing dural invasion, and perineural and arterial encasement. CT scans with multiplanar reconstructions, on the other hand, are more useful in characterizing the degree and extent of bony erosion.^2^

In 2015, Zhu et al^7^ reported 19 cases of small cell NEC of the paranasal sinuses. In this study, the authors describe a symmetrical or pigeon-like pattern in the bilateral sphenoid sinus, suggesting that this may be considered as a specific MRI feature of this subtype of tumour. In the present case, we found the same pattern on MRI (Figure 2).

There is no consensus on the treatment of sinonasal NECs.^1^ Only a small number of papers have been published in the literature, making it challenging to identify adequate treatment approaches.^6^ Pathological differentiation may not be an imperative aspect in the clinical management of this disease.^5^ Surgery, radiotherapy and chemotherapy, alone or in combination, have been suggested and used in the past.^1^ Multimodal approaches for NECs of the head and neck have been suggested in recent studies.^5^

## Learning points

1. NECs of the nasal cavity and paranasal sinuses are rare tumours, and published literature on the subject is scarce.
2. A symmetrical or pigeon-like pattern in the bilateral sphenoid sinus may be considered as a specific MRI feature of NECs in this topography, as suggested by Zhu et al.^7^

## Consent

Written informed consent was obtained from the patient for publication of this case report, including accompanying images.
